# An Observational Pilot Study on Supine Percutaneous Nephrolithotomy: Initial Experience at a Single-Centre

**DOI:** 10.7759/cureus.33597

**Published:** 2023-01-10

**Authors:** Raghubir Bhardwaj, Harish K Sinha, Ratnadip Deb, Walmik Mistari

**Affiliations:** 1 Urology, Tata Main Hospital, Jamshedpur, IND; 2 Biomedical Statistics, Tata Main Hospital, Jamshedpur, IND

**Keywords:** supine percutaneous nephrolithotomy, complication rate, stone clearance rate, renal calculi, comorbidities, supine position, percutaneous nephrolithotomy

## Abstract

Objective

To assess the surgical outcome of supine percutaneous nephrolithotomy (PCNL) in patients with co-morbidities.

Materials and methods

We retrospectively reviewed the data of 15 patients who underwent supine PCNL at our centre from September 2019 to May 2021. Preoperatively, a complete examination of the patients, along with biochemical and radiological investigations, was done. The data, which included patient demographics, comorbidities, complexity of renal calculi, complications, and stone clearance rate, were collected from the patients’ medical records.

Results

Patients aged between 31 and 70 years were included in the study. The mean (SD) BMI was 26.01 (2.31). Twelve patients (80%) were overweight with a BMI of 25.3 to 29.3 kg/m^2^. The most common comorbidities were diabetes (33.3%) and hypertension (26.7%). In our study, six patients were American Society of Anesthesiologists (ASA) grade 3 (40%), followed by grade 2 in five patients (33.3%), grade 4, and grade 1 in two patients (13.3%) each. The Guy’s Stone score was one in nine patients (60%) and two in six patients (40%). Complete clearance was achieved in 13 (86.7%) patients. Two patients (13.3%) had a stone clearance of more than 80%. Data analysis showed that 14 patients (93.3%) had no perioperative complications. Postoperative abdominal distension was noted in one patient (6.7%), which was managed conservatively (Clavien-Dindo grade 1). We did not encounter any cases of organ injury following supine PCNL. Postoperatively, none of our patients received blood transfusions.

Conclusion

Our study shows that supine PCNL is a good surgical option, especially for high-risk patients with good stone clearance and low complication rates.

## Introduction

Percutaneous nephrolithotomy (PCNL) is considered the best treatment option for large and complex renal stones, which are difficult to manage by using other treatment modalities like ureteroscopy and external shock wave lithotripsy (ESWL). This minimally invasive surgical intervention has a high stone clearance rate with low morbidity. It is also considered an effective treatment for stones in the calyceal diverticulum.

PCNL is usually performed with the patient lying prone. The prone position is considered effective, especially in large staghorn stones, but it is associated with raised intraocular pressure, circulatory difficulties [1], and restricted ventilatory capacity [2]. Moreover, it is almost impossible when a patient has certain skeletal deformities [3]. Some surgeons have started doing supine PCNL due to the high risk of complications associated with prone PCNL in certain patients.

At present, there are no guidelines regarding the approach to PCNL. This report aims to share the single-centre initial experience of supine PCNL.

## Materials and methods

A retrospective, observational study was conducted on patients who underwent supine PCNL between September 2019 and May 2022 at Tata Main Hospital (TMH), Jamshedpur. Two consultant urologists with experience in prone PCNL performed the surgery on patients with renal stones. All the patients, irrespective of their age and gender, whose renal stones needed treatment in the form of PCNL as the primary treatment were included in the study. The study excluded patients with previously operated kidneys, complex staghorn stones, and uncontrolled coagulopathies. Informed consent for the surgery was obtained from the patients.

All the patients were assessed preoperatively by proper history taking, clinical examination, and laboratory parameters including serum creatinine and urine culture. Ultrasonography of the kidney, ureter, and urinary bladder was performed by an expert radiologist. A computed tomography (CT) urography was performed to understand pelvicalyceal anatomy and the location, number, and size of the stones. The sum of all the stones was taken to determine the size of multiple stones.

Data were collected from the clinical notes and an online clinical and radiology database. Patient information like age, sex, BMI (body mass index), and American Society of Anesthesiologists (ASA) status was obtained. The case sheets provided information on comorbidities such as diabetes, hypertension, ischaemic heart disease, malignancy, and glomerular filtration rate. Data on previous renal stone treatment and urolithiasis risk factors such as hyperuricaemia and hypercalcaemia were also collected. 

The anaesthetist’s and surgeon’s notes were used to get information regarding the type of anaesthesia, surgical procedure, and stone clearance rate achieved after the procedure. Data regarding operative time, number of days in the hospital, complications (both short- and long-term), and need for blood transfusion were obtained from the hospital clinical record.

In all cases, they received preoperative antibiotics in the form of aminoglycosides or cephalosporins or according to a urine culture sensitivity report. The surgery was performed under general anaesthesia. The Galdakao-modified supine Valdivia position was used in all cases. A pelvicalyceal system (PCS) puncture was made by the operating surgeon under fluoroscopic guidance. All the punctures except one were made through the lower calyx. One puncture was made through the middle calyx. A 5F ureteric catheter was placed into the PCS. A 24F Amplatz sheath was used to extract stone fragments. In all cases, stones were fragmented by using Swiss Lithoclast Master, a combination of pneumatic and ultrasonic lithotripsy. At the end of the procedure, a Double-J (DJ) stent with or without a nephrostomy tube was used to obtain postoperative drainage.

All cases were followed up for postoperative complications and blood transfusions. Patients were discharged with a dry nephrostomy tube site and in an afebrile condition.

## Results

Fifteen supine PCNL surgeries were performed at our centre between September 2019 and May 2022. The mean (SD) age of the group was 58.33 (11.12) years, and the mean (SD) BMI was 26.01 (2.31). Twelve patients (80%) were overweight with a BMI of 25.3 to 29.3 kg/m2. Out of 15 patients, nine (60%) were men and six (40%) were women. The mean serum creatinine was 1.27 (0.60) mg/dl. Out of 15 procedures, 11 (73.3%) were performed on the left side and four (26.6%) on the right side.

**Figure 1:**
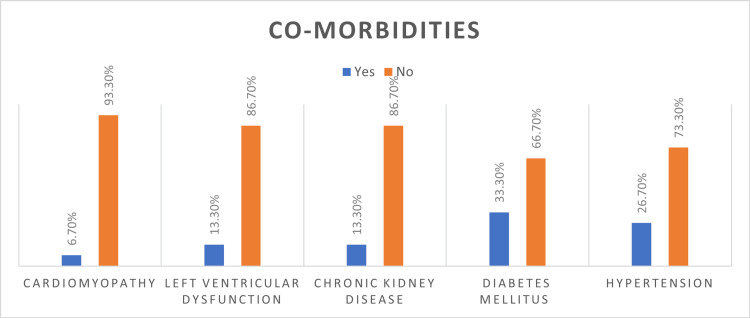
Comorbidities in our study

The most common comorbidities were diabetes (33.3%) and hypertension (26.7%). One patient had a history of cardiomyopathy (6.7%), and two patients presented with a history of left ventricular dysfunction (13.3%). Two patients had a history of chronic kidney disease (13.3%) (Figure 1).

The most common risk factors for renal calculi in our group were a history of recurrent UTI (20%) and hyperuricemia (20%). None of our patients had a history of hypercalcemia.

From an anaesthesia (ASA grade) point of view, six patients were ASA grade 3 (40%), followed by grade 2 in five patients (33.3%), grade 4, and grade 1 in two patients (13.3%) each.

When we analyzed stone location, we found that five patients (33.3%) had stones in the renal pelvis. Four patients (26.6%) in the lower calyx were followed by six patients (40%) in both the renal pelvis and lower calyx. The mean stone size was 13.9 mm. Guy’s stone score was one in nine patients (60%) and two in six patients (40%).

Complete clearance was achieved in 13 (86.7%) patients. Two patients (13.3%) had a stone clearance of more than 80%. Both patients were managed by ESWL during follow-up. Following PCNL, DJ stenting was done in all the cases (100%) and a nephrostomy tube was placed in 12 patients (80%). The DJ stenting was removed after four weeks by flexible cystoscopy as an outpatient procedure.

Data analysis showed that 14 patients (93.3%) had no perioperative complications. Postoperative abdominal distension was noted in one patient (6.7%), which was managed conservatively (Clavien-Dindo grade 1). We did not encounter any cases of organ injury following supine PCNL. Postoperatively, none of our patients received blood transfusions. The mean preoperative haemoglobin was 11.78 (1.74) g/dL, and the mean postoperative haemoglobin was 10.90 (1.72) g/dL.

The mean operative time was 91.33 (6.40) minutes, and the mean hospital stay was 5.80 (2.31 days).

## Discussion

Prone PCNL was first described by Fernstrom and Johansson [4]. Initially, there were concerns regarding an increased risk of colon injury in other positions due to the limited use of computerised tomography (CT), which gives better information regarding peri-renal anatomy. Supine PCNL, like any other operative technique, is evolving.

The mean patient age in our study of 15 patients was in their sixth decade, which is consistent with the evidence from the literature that nephrolithiasis is a disease of an ageing population. As life expectancy increases, the number of patients presenting with renal stones is likely to increase. PCNL is more difficult to perform in elderly patients due to decreased cardiopulmonary reserve and associated comorbidities [5]. The safety and efficacy of supine PCNL have already been proven.

**Figure 2:**
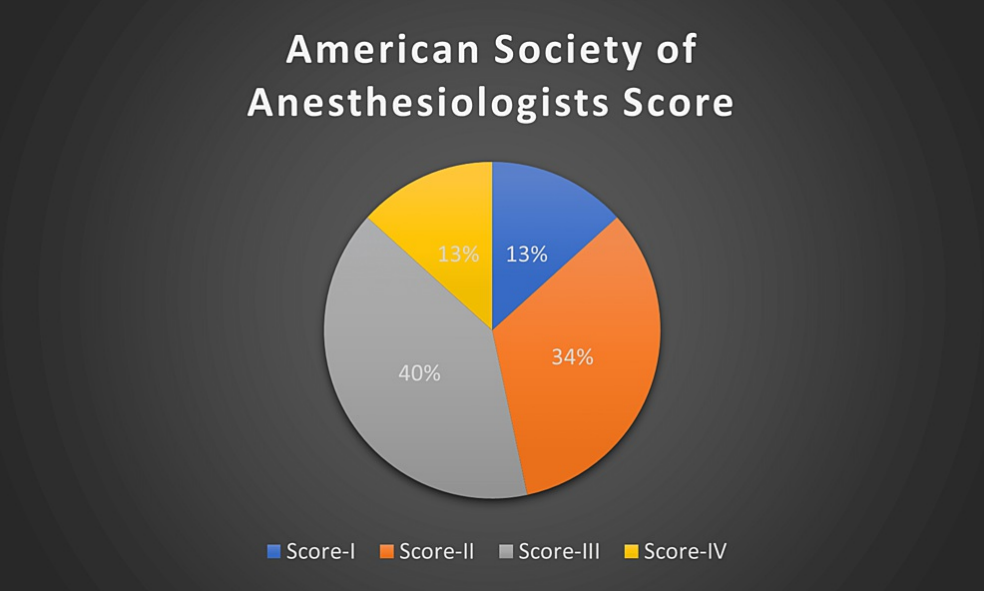
Frequency of the ASA score in our study

In our study, eight patients (53.3%) belonged to the ASA 3 and ASA 4 categories (Figure 2).

For this group of patients with significant medical issues, supine PCNL seems to be better tolerated due to less hemodynamic stress. When a patient is turned prone from a supine position, there is an increased risk of cardiovascular and respiratory compromise. When a patient is turned prone during prone PCNL, there is a risk of cervical spine injury due to excessive neck movement and musculoskeletal complications like brachial plexus injury, vocal cord compression causing hoarseness of voice, and myelic lesions [6]. There is more pressure on the eyeball and thorax and more respiratory stress in a prone position, especially in patients with a high BMI. In our study, the mean (SD) BMI was 26.01 (2.31). Twelve (80%) of the patients were overweight, with BMIs ranging from 25.2 to 29.3 kg/m2. It is easier to manage the endotracheal tube in a supine position.

We performed four (20%) tubeless PCNLs, which have the benefit of less postoperative discomfort. Supine and tubeless PCNL offer beneficial effects for both surgeries [7]. It has been shown that, in carefully selected patients, tubeless PCNL is a safe option [8]. Similarly, another meta-analysis demonstrated the safety of tubeless PCNL in selected patients [9].

In all patients (100%), a single puncture was performed. The inferior calyceal approach was used in 13 patients (86.6%), which is higher than the reports in the literature, ranging between 70.8% and 72%. Two patients (13.3%) were managed with a mid-calyx puncture. All the punctures were made under fluoroscopy guidance by the operating urologist.

In our study, the mean (SD) operative time was 91.33 (6.40) minutes (range: 80-105 minutes). Chung et al. reported a mean operative time of 78.93 +/- 3.8 minutes [10]. De Sio et al. reported a mean operative time of 43 minutes (range 25-120 minutes) [11]. The longer operative time in our study may be due to our initial experience and learning curve with supine PCNL.

Importantly, a supine position during PCNL facilitates the execution of challenging endoscopic combined intrarenal surgery [12] and simultaneous bilateral endoscopic surgery [13].

In our study, none of our patients needed a blood transfusion after supine PCNL. The mean (SD) pre-operative haemoglobin was 11.78 (1.74) g/dL, and the mean (SD) post-operative haemoglobin was 10.90 (1.72) g/dL. A lower need for blood transfusion after supine PCNL (4.3% vs. 6.1%) has been reported [14]. Similarly, a meta-analysis reported a lower incidence of blood transfusion in the supine position when compared with the prone position (weighted mean difference (WMD): 0.73; 95% confidence interval (CI): 0.56, 0.95; p = 0.02) [15]. This may be an important benefit, especially in patients with poor cardiovascular reserve.

In our study, one patient (6.7%) developed post-operative abdominal distension (Clavien-Dindo grade 1), which was managed conservatively. None of our patients required admission to the high dependency unit (HDU) postoperatively. None of our patients had a fever or sepsis-related complications after surgery, which may be due to the fact that the intrarenal pressure during the supine PCNL is low as compared to the prone PCNL. The downward position of the Amplatz sheath during supine PCNL leads to better drainage of fluids and spontaneous expulsion of stone fragments, and consequently low intrarenal pressure [16]. In our study, we found no evidence of colonic injury. The low incidence of postoperative complications is in line with the meta-analysis that confirmed the safety profile of supine PCNL [17]. Similarly, another meta-analysis showed that PCNL in a supine position could significantly reduce the rate of fever (risk ratio (RR) 0.67, 95% CI 0.46 to 0.97, p = 0.03) compared to a prone position [18].

In our study, the mean hospital stay (SD) was 5.80 (2.31) days (range: 4-13 days). A meta-analysis did not find any significant difference between the supine and prone PCNL groups (p=0.59) [18]. Similarly, another meta-analysis did not find any difference between the positions and the hospital stay (WMD: -0.14; 95% CI: -0.76; 0.47; P = 0.65) [15]. We analysed our data and found that the longer stay in our study was probably due to our hospital protocol. Our hospital is an advanced tertiary care industrial hospital that offers free treatment for all employees. Since our patients sometimes come from far-off places where satisfactory follow-up facilities are not available and there are no direct financial implications for the treatment in the hospital, we are tempted to keep them in the hospital for an extra day, especially after major surgery.

In our study, 100% stone clearance was achieved in 13 patients (86.7%) vs. nearly 80% in two patients (13.3%). Our initial results are encouraging. Similar stone clearance has been reported in supine and prone PCNL patients [19]. A meta-analysis has confirmed equivalent stone-free rates in both groups (prone and supine) of patients [20]. However, another meta-analysis showed higher stone clearance in the prone position as compared to the supine position (odds ratio (OR): 0.74; 95% CI: 0.65, 0.84; p< 0.00001) [15]. Such inconsistent data has added to the uncertainty regarding a suitable position for PCNL.

## Conclusions

In conclusion, supine PCNL is advantageous for high-risk patients with a raised BMI and cardiorespiratory disease. The supine PCNL offers anaesthetic benefits in terms of better patient control. The operating surgeon can do this procedure in a sitting position, which is more comfortable. It is a safe and effective procedure with a low complication rate, good stone clearance, and simultaneous retrograde access. There are certain limitations to our observational study. We selected relatively simple cases with a low stone burden. In addition, we did not attempt the upper-pole approach or multiple accesses. Our study did not include the paediatric population. Thus, we suggest further studies with a larger population to evaluate the benefits of supine PCNL compared to prone PCNL.
